# Association of chronic musculoskeletal pain with mortality among UK adults: A population-based cohort study with mediation analysis

**DOI:** 10.1016/j.eclinm.2021.101202

**Published:** 2021-11-14

**Authors:** Lingxiao Chen, Manuela L Ferreira, Natasha Nassar, David B Preen, John L Hopper, Shuai Li, Minh Bui, Paula R Beckenkamp, Baoyi Shi, Nigel K Arden, Paulo H Ferreira

**Affiliations:** a Faculty of Medicine and Health, Institute of Bone and Joint Research, The Kolling Institute, Northern Clinical School, University of Sydney, Sydney, NSW 2064, Australia; b Faculty of Medicine and Health, Children's Hospital Westmead Clinical School, University of Sydney, Sydney, Australia; c School of Population and Global Health, The University of Western Australia, Perth, Australia; d Centre for Epidemiology and Biostatistics, Melbourne School of Population and Global Health, University of Melbourne, Melbourne, Australia; e Centre for Cancer Genetic Epidemiology, Department of Public Health and Primary Care, University of Cambridge, Cambridge, United Kingdom; f Precision Medicine, School of Clinical Sciences At Monash Health, Monash University, Clayton, VIC, Australia; g School of Health Sciences, Faculty of Medicine and Health, University of Sydney, Sydney, Australia; h Department of Biostatistics, Mailman School of Public Health, Columbia University, NY, United States; i Nuffield Department of Orthopaedics, Rheumatology and Musculoskeletal Sciences, University of Oxford, Oxford, United Kingdom

## Abstract

**Background:**

We aimed to quantify the association between chronic musculoskeletal pain and all-cause mortality, and to investigate the extent to which this association is mediated by physical activity, smoking status, alcohol consumption, and opioid use.

**Methods:**

For this population-based cohort study, we used data from UK Biobank, UK between baseline visit (2006–2010) to 18th December 2020. We assessed the associations between chronic musculoskeletal pain and all-cause mortality using a Cox proportional hazards model. We performed causal mediation analyses to examine the proportion of the association between chronic musculoskeletal pain and all-cause mortality.

**Findings:**

Of the 384,367 included participants, a total of 187,269 participants reported chronic musculoskeletal pain. Higher number of pain sites was associated with increased risk of all-cause mortality compared to having no pain (e.g., four sites vs no site of pain, Hazard Ratio [HR] 1.46, 95% Confidence Interval [CI] 1.35 to 1.57). The multiple mediator analyses showed that the mediating proportions of all four mediators ranged from 53.4% to 122.6%: among participants with two or more pain sites, the effect estimate reduced substantially, for example, HR reduced from 1.25 (95% CI: 1.21 to 1.30; two pain sites) to 1.07 (95% CI: 1.01 to 1.11; two pain sites).

**Interpretation:**

We found that higher number of pain sites was associated with increased risk of all-cause mortality compared to having no pain, and at least half of the association of chronic musculoskeletal pain with increased all-cause mortality may be accounted for by four mediators.

**Funding:**

Twins Research Australia.

**Research in context**

**Evidence before this study**

The association between chronic musculoskeletal pain and all-cause mortality, and the extent to which this association is mediated by physical activity, smoking status, alcohol consumption, and opioid use are still unclear. Previous studies were limited by methodological limitations, including ill-defined musculoskeletal pain (e.g. pain in the last 14 days), and inappropriate comparison groups (e.g. no musculoskeletal pain within 14 days). Previous studies have not comprehensively assessed the role of lifestyle factors and certain medications (such as opioids) as possible mediators between chronic musculoskeletal pain and mortality.

**Added value of this study**

To our knowledge, this is the first large population-based study to comprehensively assess the association between chronic musculoskeletal pain (type of pain and number of pain sites) and mortality (all-cause and cause-specific mortality). Further, it is also the first study to document that the association is mediated by lifestyle factors and opioid use, individually and simultaneously. We found that higher number of pain sites was associated with increased risk of all-cause mortality compared to having no pain, and at least half of the association of chronic musculoskeletal pain with increased all-cause mortality may be accounted for by four mediators.

**Implications of all the available evidence**

This cohort study provides further evidence that higher number of pain sites was associated with increased risk of all-cause mortality compared to having no pain. However, additional evidence is needed to assess the influence from pain-related symptoms (e.g. numbness and itching) and pain duration.

Alt-text: Unlabelled box

## 1 Introduction

The global burden of chronic musculoskeletal pain is substantial, with a recent systematic review indicating a 26% prevalence of chronic musculoskeletal pain in the general adult population and 39% in those older than 65 years [1]. It is still debatable whether chronic musculoskeletal pain is associated with higher risk of mortality, due possibly to the definition of musculoskeletal pain used by previous research. For example, one Danish study addressing this question defined musculoskeletal pain as pain in the last 14 days [2]; whereas an American study defined “frequent persistent” back pain as back pain symptoms reported in the past 12 months “most of the time” or “constantly” both at baseline and first follow-up visit [3]. These definitions may not be accurately representative of a population with chronic musculoskeletal pain, because the term “chronic” is defined as pain duration of at least 3 months [4].

Another limitation in the available literature relates to selection of appropriate comparison groups. For example, the aforementioned Danish and American studies defined the comparison group as no musculoskeletal pain within 14 days, and no back pain, respectively [2,3]. Participants with non-chronic musculoskeletal pain as well as those with other types of pain (e.g., stomach or abdominal pain and pain all over the body) should be excluded from the comparison group because, failing to do so might result in the underestimation of the association between chronic musculoskeletal pain and health outcomes and/or mortality. Moreover, the co-occurrence of chronic musculoskeletal conditions is often ignored despite their great impact on the management of the index condition. For example, one previous study based on the general Dutch population indicated that more than half of the population with chronic musculoskeletal pain reported pain at two or more sites [5]. Thus, it is important to appropriately account for co-occurrence of multisite pain when assessing the association between chronic musculoskeletal pain and mortality. Finally, previous studies have not comprehensively assessed the role of lifestyle factors and certain medications (such as opioids) as possible mediators between chronic musculoskeletal pain and mortality [3,6]. Only one previous study, with a limited sample size (*n* = 6324), explored three lifestyle factors (smoking, alcohol consumption, and physical activity) individually [6]. Patients with chronic pain were more likely to smoke, be inactive and use opioid regularly [7], [8], [9]. Patients with chronic pain were less likely to drink alcohol, and this behaviour could be partly due to opioid use [10]. These modifiable factors are known to increase mortality risk [11], [12], [13], [14]. Thus, it is important to identify to what extent the association between chronic musculoskeletal pain and mortality is mediated via lifestyle factors and opioid use, when occurring individually and co-currently.

In this large prospective cohort study of middle-aged UK participants, we aimed to quantify the association between chronic musculoskeletal pain and mortality. The potential mediating roles of physical activity, smoking status, alcohol consumption, and opioid use were also explored.

## 2 Methods

### 2.1 Data

This study used data from the UK Biobank which recruited approximately 500,000 people aged 40–69 years between 2006 and 2010, from 22 centres in the UK [15]. This study was restricted to a subset at the initial assessment (2006–2010): participants with chronic musculoskeletal pain (neck or shoulder pain, back pain, hip pain, and knee pain) represented the exposed group, and those without pain made up the comparison group. Participants who experienced headache, facial pain, stomach or abdominal pain or pain all over the body were excluded. Participants who experienced musculoskeletal pain in the last month but did not report they ever had chronic musculoskeletal pain were also excluded. Details of the UK Biobank can be found in the registry online protocol: http://www.ukbiobank.ac.uk. The North West Multi-centre Ethics Committee granted ethical approval to access data from the UK Biobank, and all participants provided written informed consent. We report this study based on the Strengthening the Reporting of Observational Studies in Epidemiology (STROBE) statement [16]. The study was conducted under UK Biobank project number 56,837.

### 2.2 Exposure

As our study focused on musculoskeletal pain given it is a major contributor to the global burden of disease [17]. Musculoskeletal pain was defined using the options in the UK Biobank touchscreen questionnaire (Category 100,048) which includes headache, facial pain, neck or shoulder pain, back pain, stomach or abdominal pain, hip pain, knee pain and pain all over the body. Headache, facial pain, pain all over the body, and stomach or abdominal pain were not included in defining the exposure as there was insufficient evidence these were related to musculoskeletal conditions and were therefore beyond the scope of this study. Thus, chronic musculoskeletal pain was defined by responses of participants to two questions: 1. “In the last month have you experienced any of the following that interfered with your usual activities?”; 2. “Have you had neck or shoulder pains/back pains/hip pains/knee pains for more than 3 months?”. Participants who answered yes to both questions were defined as participants who had chronic musculoskeletal pain. Considering the co-occurrence of chronic musculoskeletal pain conditions, we have divided the exposure into two parts: 1. type of pain for those with one pain site as: neck or shoulder pain only, back pain only, hip pain only and knee pain only; 2. number of pain sites as: one, two, three, or four pain sites. As question 2 follows question 1, the comparison group was composed by those who answered ‘none of the above’ to question 1. This is because if participants indicated they did not experience back pain in the last month that interfered with their usual activities (question 1), they would not be asked question 2: "Have you had back pains for more than 3 months?".

### 2.3 Outcome

Follow-up was ascertained from baseline, i.e., initial assessment visit when chronic musculoskeletal pain was measured (2006–2010); and continued until death was confirmed via the death registry, the participant withdrew from the study, or until the end of the follow-up period on 18th December 2020, whichever came first. The primary outcome was all-cause mortality. The secondary outcome was cause-specific mortality which was identified from underlying (primary) cause of death in the death registry. Based on clinical knowledge and the large sample available in the UK Biobank, cause-specific mortality was defined as cancer (International Classification of Diseases 10th edition [ICD-10] codes C00 to C97), cardiovascular disease (ICD-10 codes I05 to I89), mental and behavioural disorder (ICD-10 codes F00 to F89), respiratory system disease (ICD-10 codes J09 to J99), suicide (ICD-10 codes X60 to X84), nervous system disease (ICD-10 codes G00 to G99), endocrine, nutritional and metabolic disease (ICD-10 codes E00 to E90), digestive system disease (ICD-10 codes K20 to K93), musculoskeletal system and connective tissue disease (ICD-10 codes M00 to M90), genitourinary system disease (ICD-10 codes N00 to N98), falls (ICD-10 codes W00 to W19), and others (remaining ICD-10 codes). We followed the ICD-10 definitions of death causes of morbidity and mortality and examined the outcomes ‘death due to mental and behavioural disorders’ (i.e. Chapter V Mental and behavioural disorders) and ‘suicide’ (i.e. Chapter XX External), separately.

### 2.4 Mediators

Physical activity, smoking status, alcohol consumption, and opioid use were included as potential mediators. These measures were assessed at the initial visit (2006–2010). Based on one previous study, physical activity participation was assessed using the International Physical Activity Questionnaire (IPAQ) activity group (low, <10.0; moderate, 10.0–49.9 and high, >=50 excess metabolic equivalent (MET)-hours/week) [18]. We used the data from the IPAQ activity group (Data-Field 22,032). The calculation methods could be found from the previous study [18]*.* The data was generated as part of UKB Application ID 12,184. The mediator of physical activity was modelled as low vs moderate or high. Based on one previous study, alcohol consumption was measured as alcohol intake frequency (daily or almost daily, three or four times a week, once or twice a week, one to three times a month, special occasions only and never; regular referred to the first three categories) [19]. The mediator of alcohol consumption was modelled as regular vs special occasions or never. Smoking status was defined as ‘never’, ‘previous smoking’, and ‘current smoking’. The mediator of smoking status was modelled as current smoking vs never or previous smoking. Opioid use was defined using the regular medication use question (detailed names and codes can be found in Appendix S1). This questionnaire (Category 20,003) contains data on any regular treatments taken weekly, monthly, etc. (without doses and formulations). The mediator of opioid use was modelled as yes vs no. Two types of multiple mediators were created: one focused on lifestyle factors including physical activity, smoking status, and alcohol consumption; and the other combining all four.

### 2.5 Covariates

To avoid potential overadjustment, factors known to be associated with both chronic musculoskeletal pain and mortality as well as those occurring before chronic musculoskeletal pain was reported, were included as confounders [20]. These variables included age, sex, ethnicity, and the Townsend deprivation index [21]. Age was defined as a continuous variable. Sex was defined as a binary variable (female vs male). Ethnicity was defined as an unordered categorical variable (white, black, Asian, mixed, and other). Townsend deprivation index was defined as a continuous variable. The Townsend deprivation index is a composite measure of deprivation based on unemployment, non-car ownership, non-home ownership, and household overcrowding; a negative value represents high socioeconomic status. Each participant is assigned a score corresponding to the output area in which their postcode is located.

### 2.6 Statistical analysis

Descriptive statistics were used to describe the baseline characteristics (e.g. number of pain site, pain type, race/ethnicity, age, sex, Townsend deprivation index, body mass index, smoking status, alcohol consumption, physical activity, opioid use, mental wellbeing, and comorbidity) among participants with chronic musculoskeletal pain and the comparison group. We examined the association between chronic musculoskeletal pain and all-cause mortality using Cox proportional hazards regression models [22]. We established a stepped modelling framework: step 1, unadjusted analyses; and step 2, analyses adjusted for age, sex, ethnicity, and the Townsend deprivation index. Results from model 2 are reported in the results section. Hazard ratios (HRs) with 95% confidence intervals (CIs) were calculated. We firstly checked the proportional hazards (pH) assumption through goodness-of-fit test (cox.zph function from survival package) [23]. If any significant result was found, we then graphically assessed pH assumption through log-log Kaplan Meier plot [24]. Overall, the pH assumption was met. Chronic musculoskeletal pain was modelled with the type of pain and number of pain sites as described above. For the analysis of the type of pain, the exposure was examined using unordered categorical variables. For the analysis on the number of pain sites, we treated the number of pain sites as an unordered categorical variable initially and then performed a trend analysis (the number of pain sites was treated as a continuous variable) [20]. Results of the trend analysis are presented in Table 3. Cause-specific mortality was modelled through multistate survival model with the calculation of transition probability to account for competing risk of death due to other causes [25]. The above-mentioned stepped modelling framework was used. Complete case analysis (i.e. excluding participants with missing data in any included variable) was used for the main analysis given the percentage of missing data was negligible (e.g., 0.1% for Townsend deprivation index) [26]. The strategy to handle missing data in causal mediation analysis is listed below.

We performed causal mediation analyses to examine the proportion of the association between chronic musculoskeletal pain and all-cause mortality mediated by physical activity, smoking status, alcohol consumption, and opioid use [27]. We assumed the existence of potential interactions between the exposure and the mediator; and used regression-based approaches which allowed for the existence of exposure-mediator interaction to estimate the total effect, total natural indirect effect (TNIE) and total natural direct effect (TNDE) [27]. The TNIE represented the effect of chronic musculoskeletal pain on all-cause mortality that could be explained by its association with the inclusion of the mediator/s in the model. The TNDE represented the effect of chronic musculoskeletal pain on all-cause mortality that was independent of the mediator. The proportion of the association by the mediator (TNIE/[TNDE + TNIE]) was estimated to quantify the magnitude of mediation. Considering the missing data issue in some mediators (19.6% for physical activity, 0.4% for smoking status and 0.08% for alcohol consumption), bootstrap with multiple imputation was used to obtain robust HRs with 95% CIs.

For the primary outcome, several exploratory and sensitivity analyses were performed to confirm the robustness of the results (details could be found in Appendix S2). We examined whether the association between the exposure and all-cause mortality differed by sex, age, BMI, ethnicity, or smoking status through testing of multiplicative interactions using WALD statistics [22]. We used lag period analysis (excluding events which occur within 3-month, 6-month, 1-year, 3-year, 5-year and 7-year after enrolment) to verify any potential induction period (exposure status at a given time will correlate with a possible increase or decrease in disease only at some later time), inverse probability treatment weighting with covariates which might be considered as confounders (e.g. depression and anxiety) to identify potential model misspecification, excluding participants with cancer at baseline given these death might be less likely to be caused primarily by musculoskeletal pain, and e-value to test for unmeasured confounding [20]. All statistical analyses were performed with tidyverse, rms, hmisc, survival, etm, and CMAverse [28] packages in R, version 4.04 (R Group for Statistical Computing). LC and PHF had access to the data.

## 3 Role of the funding source

The funding source had no role in the design and conduct of the study; collection, management, analysis, and interpretation of the data; preparation, review, or approval of the manuscript; and decision to submit the manuscript for publication.

## 4 Results

### 4.1 Summary

**Fig. 1:**
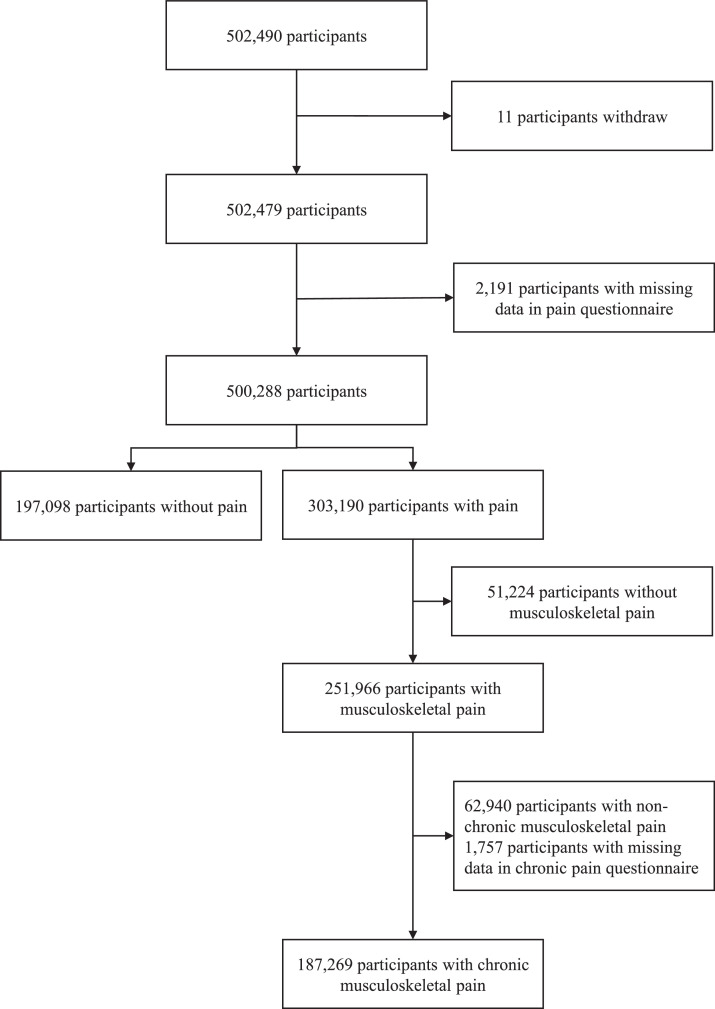
Flow chart. The reasons for ineligibility and the numbers of ineligible participants were shown on the right arrow. The numbers of potential eligible participants were connected through the down arrow.

Of the 384,367 included participants (Fig. 1), 208,412 (54.2%) were women, and the mean (SD) age was 57 (8) years (Table 1). A total of 187,269 participants reported chronic musculoskeletal pain: more than half reported one pain site (*n* = 112,227, 59.9%), followed by two (*n* = 49,126, 26.2%), three (*n* = 19,107, 10.2%) and four pain sites (6809, 3.6%). About one fifth reported knee pain only (*n* = 37,002, 19.8%), followed by back pain only (*n* = 33,731, 18.0%), neck or shoulder pain only (*n* = 31,331, 16.7%) and hip pain only (*n* = 10,163, 5.4%). Table 1 presents the participants’ characteristics.

**Table 1:** Participant characteristics at the UK Biobank assessment.

**Characteristic**	**No pain(*n*** **=** **197,098)**	**Chronic pain(*n*** **=** **187,269)**	**Total sample(*n*** **=** **384,367)**
Number of pain site			
One	NA	112,227 (59.9)	112,227 (29.2)
Two	NA	49,126 (26.2)	49,126 (12.8)
Three	NA	19,107 (10.2)	19,107 (5.0)
Four	NA	6809 (3.6)	6809 (1.8)
Pain type			
Neck or shoulder pain only	NA	31,331 (16.7)	31,331 (8.2)
Back pain only	NA	33,731 (18.0)	33,731 (8.8)
Hip pain only	NA	10,163 (5.4)	10,163 (2.6)
Knee pain only	NA	37,002 (19.8)	37,002 (9.6)
Mixed	NA	75,042 (40.1)	75,042 (19.5)
Race/ethnicity			
White	188,601 (95.7)	175,872 (93.9)	364,473 (94.8)
Black	2286 (1.2)	3221 (1.7)	5507 (1.4)
Asian	2707 (1.4)	4009 (2.1)	6716 (1.7)
Mixed	965 (0.5)	1169 (0.6)	2134 (0.6)
Other	2539 (1.3)	2998 (1.6)	5537 (1.4)
Age	56.7 (8.1)	57.2 (7.9)	57.0 (8.0)
Male	93,471 (47.4)	82,484 (44.1)	175,955 (45.8)
Townsend deprivation index, mean (SD)	−1.6 (2.9)	−1.1 (3.2)	−1.3 (3.1)
Missing	218 (0.1)	256 (0.1)	474 (0.1)
Body mass index, mean (SD)	26.7 (4.3)	28.3 (5.1)	27.5 (4.8)
Missing	781 (0.4)	1163 (0.6)	1944 (0.5)
Smoking status			
Current	17,401 (8.8)	22,776 (12.2)	40,177 (10.5)
Previous	65,866 (33.4)	69,266 (37.0)	135,132 (35.2)
Never	113,239 (57.5)	94,421 (50.4)	207,660 (54.0)
Missing	592 (0.3)	806 (0.4)	1398 (0.4)
Alcohol consumption			
Daily or almost daily	44,251 (22.5)	36,389 (19.4)	80,640 (21.0)
3–4 times a week	49,641 (25.2)	40,085 (21.4)	89,726 (23.3)
1–2 times a week	51,366 (26.1)	47,088 (25.1)	98,454 (25.6)
Unregular or never	51,725 (26.2)	63,517 (33.9)	115,242 (30.0)
Missing	115 (0.06)	190 (0.1)	305 (0.08)
Physical activity			
Low	27,662 (14.0)	30,101 (16.1)	57,763 (15.0)
Moderate	66,768 (33.9)	58,845 (31.4)	125,613 (32.7)
High	67,628 (34.3)	58,021 (31.0)	125,649 (32.7)
Missing	35,040 (17.8)	40,302 (21.5)	75,342 (19.6)
Opioid use	1771 (0.9)	18,811 (10.0)	20,582 (5.4)
Mental Wellbeing			
Depression	10,990 (5.6)	14,183 (7.6)	25,173 (6.5)
Anxiety	7417 (3.8)	9093 (4.9)	16,510 (4.3)
Comorbidity			
Diabetes	8599 (4.4)	12,044 (6.4)	20,643 (5.4)
Missing	324 (0.2)	673 (0.4)	997 (0.3)
Cancer	14,815 (7.5)	15,279 (8.2)	30,094 (7.8)
Missing	397 (0.2)	741 (0.4)	1138 (0.3)
Cardiovascular disease^a^			
One	47,047 (23.9)	55,778 (29.8)	102,825 (26.8)
Two	3811 (1.9)	7025 (3.8)	10,836 (2.8)
Three	778 (0.4)	1951 (1.0)	2729 (0.7)
Four	57 (0.0)	224 (0.1)	281 (0.1)

Data are presented as number (percentage) of patients unless otherwise indicated. For opioid use, depression and anxiety, participants who did not report them were treated as no use or no disease. For cardiovascular disease, we treated missing data as no disease to facilitate the calculation of the number of cardiovascular disease. We think it is fine because the percentage of missing data in the question - vascular/heart problems diagnosed by doctor (Data-Field 6150) is tiny (<0.3%).

a Four types of cardiovascular disease were included: heart attack, angina, stroke, high blood pressure.

### 4.2 All-cause mortality

There were 25,917 deaths recorded over a mean follow-up time of 7.4 years (SD: 3.3, range: 0.0 to 14.6 years). Compared with participants without pain, the multivariable adjusted HR for all-cause mortality was 1.07 (95% CI 1.02 to 1.13) for participants with neck or shoulder pain only, 1.17 (95% CI 1.11 to 1.22) for participants with back pain only, 1.15 (95% CI 1.07 to 1.24) for participants with hip pain only and 1.03 (95% CI 0.99 to 1.08) for participants with knee pain only (Table 2). Participants with one (HR 1.09, 95% CI 1.06 to 1.12), two (HR 1.25, 95% CI 1.21 to 1.30), three (HR 1.43, 95% CI 1.36 to 1.51) and four (HR 1.46, 95% CI 1.35 to 1.57) pain sites had an increased risk of all-cause mortality (Table 2) compared to those without pain. Exploratory analyses (Appendix S3) indicated that for participants with two or more pain sites, younger age (<65 years) was associated with higher risk of all-cause mortality compared to older age with the same number of sites. The results from the sensitivity analyses (Appendix S4–S7) were similar to those of the main analyses (e.g. four pain sites vs no pain: original, HR 1.46, 95% CI 1.35 to 1.57; excluding participants who died within 3 months after enrolment, HR 1.46, 95% CI 1.35; excluding participants with cancer at baseline, HR 1.49, 95% CI 1.37 to 1.63).

**Table 2:** Hazard Ratios (95% Confidence Intervals) for mortality according to pain type.

Cause of death	No pain(*n* = 197,098)	Neck or shoulder pain only (*n* = 31,331)	Back pain only(*n* = 33,731)	Hip pain only(*n* = 10,163)	Knee pain only(*n* = 37,002)
**All cause**					
No of deaths (*n* = 19,441)	11,877	1957	2305	769	2533
Risk of death,%	6.0	6.2	6.8	7.6	6.8
Unadjusted	1 (reference)	1.03 (0.98, 1.08)	1.14 (1.09, 1.19)	1.25 (1.16, 1.34)	1.14 (1.10, 1.19)
Multivariable adjusted^a^	1 (reference)	1.07 (1.02, 1.13)	1.17 (1.11, 1.22)	1.15 (1.07, 1.24)	1.03 (0.99, 1.08)
**Cancer**					
No of deaths (*n* = 10,363)	6502	1000	1172	388	1301
Risk of death,%	3.3	3.2	3.5	3.8	3.5
Unadjusted	1 (reference)	0.96 (0.90, 1.03)	1.05 (0.99, 1.12)	1.16 (1.05, 1.29)	1.07 (1.01, 1.14)
Multivariable adjusted^a^	1 (reference)	1.00 (0.94, 1.07)	1.10 (1.03, 1.17)	1.06 (0.96, 1.18)	0.99 (0.93, 1.05)
**Endocrine, nutritional and metabolic disease**					
No of deaths (*n* = 196)	109	34	26	8	19
Risk of death,%	0.06	0.1	0.08	0.08	0.05
Unadjusted	1 (reference)	1.95 (1.33, 2.86)	1.39 (0.91, 2.14)	1.44 (0.70, 2.94)	0.94 (0.58, 1.52)
Multivariable adjusted^a^	1 (reference)	1.99 (1.35, 2.93)	1.34 (0.87, 2.05)	1.39 (0.68, 2.86)	0.81 (0.50, 1.33)
**Mental and behavioural disorder**					
No of deaths (*n* = 410)	239	40	62	13	56
Risk of death,%	0.1	0.1	0.2	0.1	0.2
Unadjusted	1 (reference)	1.04 (0.74, 1.45)	1.51 (1.14, 2.00)	1.06 (0.61, 1.86)	1.26 (0.94, 1.69)
Multivariable adjusted^a^	1 (reference)	1.09 (0.78, 1.52)	1.58 (1.19, 2.09)	0.92 (0.52, 1.61)	1.09 (0.81, 1.46)
**Nervous system disease**					
No of deaths (*n* = 1048)	649	110	120	42	127
Risk of death,%	0.3	0.4	0.4	0.4	0.3
Unadjusted	1 (reference)	1.05 (0.86, 1.29)	1.08 (0.89, 1.31)	1.26 (0.93, 1.73)	1.05 (0.87, 1.27)
Multivariable adjusted^a^	1 (reference)	1.11 (0.91, 1.36)	1.16 (0.95, 1.40)	1.12 (0.82, 1.54)	0.96 (0.80, 1.16)
**Cardiovascular disease**					
No of deaths (*n* = 3728)	2245	377	436	163	507
Risk of death,%	1.1	1.2	1.3	1.6	1.4
Unadjusted	1 (reference)	1.05 (0.94, 1.17)	1.14 (1.02, 1.26)	1.42 (1.21, 1.66)	1.21 (1.10, 1.33)
Multivariable adjusted^a^	1 (reference)	1.12 (1.00, 1.25)	1.15 (1.04, 1.28)	1.35 (1.15, 1.58)	1.07 (0.97, 1.17)
**Respiratory system disease**					
No of deaths (*n* = 1290)	720	151	183	67	169
Risk of death,%	0.4	0.5	0.5	0.7	0.5
Unadjusted	1 (reference)	1.31 (1.10, 1.56)	1.49 (1.26, 1.75)	1.82 (1.42, 2.34)	1.26 (1.06, 1.49)
Multivariable adjusted^a^	1 (reference)	1.35 (1.13, 1.61)	1.49 (1.26, 1.75)	1.63 (1.27, 2.10)	1.08 (0.91, 1.28)
**Digestive system disease**					
No of deaths (*n* = 674)	382	74	84	21	113
Risk of death,%	0.2	0.2	0.2	0.2	0.3
Unadjusted	1 (reference)	1.21 (0.94, 1.55)	1.29 (1.02, 1.63)	1.07 (0.69, 1.66)	1.59 (1.29, 1.96)
Multivariable adjusted^a^	1 (reference)	1.26 (0.98, 1.62)	1.28 (1.01, 1.62)	1.03 (0.67, 1.60)	1.42 (1.15, 1.76)
**Musculoskeletal system and connective tissue disease**					
No of deaths (*n* = 64)	34	7	11	1	11
Risk of death,%	0.02	0.02	0.03	0.01	0.03
Unadjusted	1 (reference)	1.28 (0.57, 2.89)	1.89 (0.96, 3.73)	0.57 (0.08, 4.19)	1.73 (0.88, 3.42)
Multivariable adjusted^a^	1 (reference)	1.30 (0.57, 2.93)	1.89 (0.95, 3.74)	0.52 (0.07, 3.79)	1.55 (0.79, 3.07)
**Genitourinary system disease**					
No of deaths (*n* = 109)	62	14	14	4	15
Risk of death,%	0.03	0.04	0.04	0.04	0.04
Unadjusted	1 (reference)	1.41 (0.79, 2.52)	1.32 (0.74, 2.36)	1.26 (0.46, 3.46)	1.30 (0.74, 2.28)
Multivariable adjusted^a^	1 (reference)	1.39 (0.78, 2.49)	1.33 (0.74, 2.38)	1.09 (0.40, 3.01)	1.03 (0.58, 1.85)
**Falls**					
No of deaths (*n* = 138)	88	10	21	3	16
Risk of death,%	0.04	0.03	0.06	0.03	0.04
Unadjusted	1 (reference)	0.71 (0.37, 1.37)	1.40 (0.87, 2.25)	0.66 (0.21, 2.10)	0.97 (0.57, 1.66)
Multivariable adjusted^a^	1 (reference)	0.73 (0.38, 1.41)	1.41 (0.87, 2.27)	0.60 (0.19, 1.90)	0.84 (0.49, 1.43)
**Suicide**					
No of deaths (*n* = 155)	103	14	19	4	15
Risk of death,%	0.05	0.04	0.06	0.04	0.04
Unadjusted	1 (reference)	0.85 (0.49, 1.49)	1.08 (0.66, 1.76)	0.76 (0.28, 2.05)	0.78 (0.45, 1.34)
Multivariable adjusted^a^	1 (reference)	0.90 (0.51, 1.57)	1.00 (0.61, 1.63)	0.87 (0.32, 2.36)	0.77 (0.45, 1.32)
**Other**					
No of deaths (*n* = 1257)	744	126	157	46	184
Risk of death,%	0.4	0.4	0.5	0.5	0.5
Unadjusted	1 (reference)	1.05 (0.87, 1.27)	1.23 (1.04, 1.46)	1.21 (0.90, 1.63)	1.33 (1.13, 1.56)
Multivariable adjusted^a^	1 (reference)	1.07 (0.89, 1.30)	1.21 (1.02, 1.44)	1.14 (0.85, 1.54)	1.18 (1.00, 1.39)

a Adjusted for age, sex, townsend deprivation index and ethnicity.

### 4.3 Cause-specific mortality

The most common cause of death was cancer (13,488, 52.0%), followed by cardiovascular disease (5021, 19.4%) and respiratory system disease (1855, 7.2%) (Table 3). For participants with neck or shoulder pain only, there was a strong positive association with mortality related to endocrine, nutritional and metabolic disease (HR 1.99, 95% CI 1.35 to 2.93), and respiratory system disease (HR 1.35, 95% CI 1.13 to 1.61). Back pain only was associated with increased risk of mortality related to mental and behavioural disorder (HR 1.58, 95% CI 1.19 to 2.09), and respiratory system disease (HR 1.49, 95% CI 1.26 to 1.75). Mortality related to cardiovascular disease (HR 1.35, 95% CI 1.15 to 1.58), and respiratory system disease (HR 1.63, 95% CI 1.27 to 2.10) was more likely in participants with hip pain only, while people with knee pain only had greater risk of mortality from diseases of the digestive system (HR 1.42, 95% CI 1.15 to 1.76). Participants with two or more pain sites had a higher risk of death from respiratory system disease, and digestive system disease, with hazard ratios ranging from 1.29 to 2.50. Appendix S8 lists transition probabilities (e.g. whole cohort; baseline to death due to cancer 3.5%; baseline to death due to cardiovascular disease 1.3%).

**Table 3:** Hazard Ratios (95% Confidence Intervals) for mortality according to number of pain sites.

Cause of death	No pain(*n* = 197,098)	One(*n* = 112,227)	Two(*n* = 49,126)	Three(*n* = 19,107)	Four(*n* = 6809)	Trend analysis(*n* = 384,367)
**All cause**						
No of deaths (*n* = 25,917)	11,877	7555	3949	1847	689	25,917
Risk of death,%	6.0	6.7	8.0	9.7	10.1	6.7
Unadjusted	1 (reference)	1.12 (1.09, 1.15)	1.34 (1.29, 1.39)	1.62 (1.54, 1.70)	1.69 (1.57, 1.83)	1.16 (1.15, 1.17)
Multivariable adjusted^a^	1 (reference)	1.09 (1.06, 1.12)	1.25 (1.21, 1.30)	1.43 (1.36, 1.51)	1.46 (1.35, 1.57)	1.12 (1.10, 1.13)
**Cancer**						
No of deaths (*n* = 13,488)	6502	3861	1975	859	291	13,488
Risk of death,%	3.3	3.4	4.0	4.5	4.3	3.5
Unadjusted	1 (reference)	1.04 (1.00, 1.09)	1.22 (1.16, 1.29)	1.38 (1.28, 1.48)	1.31 (1.16, 1.47)	1.10 (1.08, 1.11)
Multivariable adjusted^a^	1 (reference)	1.03 (0.99, 1.07)	1.15 (1.10, 1.21)	1.24 (1.15, 1.33)	1.15 (1.03, 1.30)	1.06 (1.04, 1.08)
**Endocrine, nutritional and metabolic disease**						
No of deaths (*n* = 295)	109	87	51	37	11	295
Risk of death,%	0.06	0.08	0.1	0.2	0.2	0.08
Unadjusted	1 (reference)	1.40 (1.06, 1.86)	1.89 (1.36, 2.64)	3.55 (2.44, 5.15)	2.96 (1.59, 5.50)	1.42 (1.28, 1.56)
Multivariable adjusted^a^	1 (reference)	1.32 (1.00, 1.75)	1.69 (1.21, 2.36)	2.91 (2.00, 4.27)	2.29 (1.23, 4.28)	1.33 (1.21, 1.47)
**Mental and behavioural disorder**						
No of deaths (*n* = 543)	239	171	77	43	13	543
Risk of death,%	0.1	0.2	0.2	0.2	0.2	0.1
Unadjusted	1 (reference)	1.26 (1.03, 1.53)	1.30 (1.00, 1.68)	1.87 (1.35, 2.59)	1.59 (0.91, 2.77)	1.17 (1.08, 1.27)
Multivariable adjusted^a^	1 (reference)	1.21 (0.99, 1.47)	1.17 (0.90, 1.52)	1.60 (1.16, 2.22)	1.35 (0.77, 2.36)	1.12 (1.03, 1.21)
**Nervous system disease**						
No of deaths (*n* = 1388)	649	399	202	102	36	1388
Risk of death,%	0.3	0.4	0.4	0.5	0.5	0.4
Unadjusted	1 (reference)	1.08 (0.95, 1.22)	1.25 (1.07, 1.47)	1.64 (1.33, 2.02)	1.62 (1.16, 2.26)	1.14 (1.09, 1.20)
Multivariable adjusted^a^	1 (reference)	1.07 (0.95, 1.22)	1.20 (1.02, 1.40)	1.51 (1.23, 1.87)	1.50 (1.07, 2.10)	1.12 (1.06, 1.18)
**Cardiovascular disease**						
No of deaths (*n* = 5021)	2245	1483	785	353	155	5021
Risk of death,%	1.1	1.3	1.6	1.8	2.3	1.3
Unadjusted	1 (reference)	1.16 (1.09, 1.24)	1.41 (1.30, 1.53)	1.64 (1.46, 1.83)	2.01 (1.71, 2.37)	1.19 (1.15, 1.22)
Multivariable adjusted^a^	1 (reference)	1.12 (1.05, 1.20)	1.31 (1.21, 1.43)	1.45 (1.29, 1.62)	1.73 (1.47, 2.04)	1.14 (1.11, 1.17)
**Respiratory system disease**						
No of deaths (*n* = 1855)	720	570	313	173	79	1855
Risk of death,%	0.4	0.5	0.6	0.9	1.2	0.5
Unadjusted	1 (reference)	1.39 (1.25, 1.55)	1.75 (1.54, 2.00)	2.51 (2.12, 2.96)	3.21 (2.54, 4.05)	1.34 (1.29, 1.40)
Multivariable adjusted^a^	1 (reference)	1.32 (1.18, 1.48)	1.57 (1.37, 1.79)	2.07 (1.75, 2.44)	2.50 (1.98, 3.16)	1.26 (1.21, 1.31)
**Digestive system disease**						
No of deaths (*n* = 967)	382	292	177	84	32	967
Risk of death,%	0.2	0.3	0.4	0.4	0.5	0.3
Unadjusted	1 (reference)	1.34 (1.15, 1.56)	1.87 (1.56, 2.23)	2.29 (1.81, 2.90)	2.45 (1.71, 3.51)	1.31 (1.24, 1.38)
Multivariable adjusted^a^	1 (reference)	1.29 (1.11, 1.51)	1.73 (1.44, 2.06)	1.98 (1.56, 2.51)	1.97 (1.37, 2.85)	1.25 (1.18, 1.32)
**Musculoskeletal system and connective tissue disease**						
No of deaths (*n* = 94)	34	30	17	11	2	94
Risk of death,%	0.02	0.03	0.03	0.06	0.03	0.02
Unadjusted	1 (reference)	1.55 (0.95, 2.53)	2.01 (1.12, 3.60)	3.36 (1.70, 6.64)	1.71 (0.41, 7.13)	1.36 (1.14, 1.62)
Multivariable adjusted^a^	1 (reference)	1.50 (0.92, 2.46)	1.84 (1.03, 3.31)	2.90 (1.46, 5.75)	1.42 (0.34, 5.92)	1.30 (1.09, 1.55)
**Genitourinary system disease**						
No of deaths (*n* = 159)	62	47	27	17	6	159
Risk of death,%	0.03	0.04	0.06	0.09	0.09	0.04
Unadjusted	1 (reference)	1.33 (0.91, 1.95)	1.76 (1.12, 2.76)	2.86 (1.67, 4.89)	2.83 (1.22, 6.54)	1.35 (1.18, 1.55)
Multivariable adjusted^a^	1 (reference)	1.22 (0.83, 1.79)	1.51 (0.96, 2.38)	2.24 (1.30, 3.84)	2.08 (0.89, 4.82)	1.25 (1.09, 1.44)
**Falls**						
No of deaths (*n* = 178)	88	50	28	7	5	178
Risk of death,%	0.04	0.04	0.06	0.04	0.07	0.05
Unadjusted	1 (reference)	1.00 (0.71, 1.41)	1.28 (0.84, 1.96)	0.83 (0.38, 1.79)	1.66 (0.67, 4.08)	1.06 (0.92, 1.23)
Multivariable adjusted^a^	1 (reference)	0.95 (0.67, 1.34)	1.15 (0.75, 1.76)	0.69 (0.32, 1.49)	1.31 (0.53, 3.24)	1.00 (0.87, 1.16)
**Suicide**						
No of deaths (*n* = 196)	103	52	22	11	8	196
Risk of death,%	0.05	0.05	0.04	0.06	0.1	0.05
Unadjusted	1 (reference)	0.89 (0.64, 1.24)	0.86 (0.54, 1.37)	1.11 (0.60, 2.07)	2.27 (1.11, 4.66)	1.06 (0.92, 1.22)
Multivariable adjusted^a^	1 (reference)	0.89 (0.64, 1.24)	0.90 (0.57, 1.43)	1.18 (0.63, 2.20)	2.47 (1.20, 5.10)	1.08 (0.94, 1.24)
**Other**						
No of deaths (*n* = 1733)	744	513	275	150	51	1733
Risk of death,%	0.4	0.5	0.6	0.8	0.7	0.5
Unadjusted	1 (reference)	1.21 (1.08, 1.35)	1.49 (1.29, 1.71)	2.09 (1.76, 2.50)	1.99 (1.50, 2.64)	1.23 (1.18, 1.29)
Multivariable adjusted^a^	1 (reference)	1.16 (1.04, 1.30)	1.36 (1.18, 1.56)	1.80 (1.51, 2.15)	1.65 (1.24, 2.20)	1.18 (1.13, 1.23)

a Adjusted for age, sex, townsend deprivation index and ethnicity.

### 4.4 Mediation analyses

Table 4 presents the total, direct and indirect associations of chronic musculoskeletal pain with all-cause mortality as well as the proportion mediated. The single mediator analyses showed the following mediating proportions of the association between chronic musculoskeletal pain and all-cause mortality: 8.0 to 15.7% for physical activity; 32.5 to 79.0% for opioid use; 14.6 to 29.8% for smoking status and 2.4 to 17.5% for alcohol consumption. The multiple mediator analyses showed that, combined, the effect of the lifestyle factors ranged from 30.5 to 42.8%, and all four mediators ranged from 53.4 to 122.6%: for participants with one pain site, chronic musculoskeletal pain was not associated with all-cause mortality, for example, for people with back pain only, HR 1.04, 95% CI 0.99 to 1.08 vs original, HR 1.17, 95% CI 1.11 to 1.22; for participants with two or more pain sites, the effect estimate presented a greater reduction, for example, HR reduced from 1.25 (95% CI 1.21 to 1.30; two pain sites) to 1.07 (95% CI 1.01 to 1.11; two pain sites).

**Table 4:** Mediation analysis for the association between pain Type, number of pain sites, and all-cause mortality.

**Mediators**	**Pain type**			
	**Neck or shoulder pain only**	**Back pain only**	**Hip pain only**	**Knee pain only**
Total association^a^	**1.08 (1.05, 1.12)**	**1.17 (1.11, 1.22)**	**1.16 (1.08, 1.24)**	1.03 (0.98, 1.07)
**Opioid use**^b^				
Natural direct association	1.03 (0.97, 1.06)	**1.08 (1.03, 1.12)**	1.09 (0.99, 1.15)	0.98 (0.94, 1.03)
Natural indirect association	**1.03 (1.02, 1.05)**	**1.07 (1.05, 1.09)**	**1.05 (1.03, 1.08)**	**1.03 (1.02, 1.04)**
Proportion mediated,%	42.6	45.2	34.9	78.9
**Smoking status**^c^				
Natural direct association	**1.06 (1.01, 1.10)**	**1.13 (1.06, 1.18)**	**1.12 (1.05, 1.21)**	1.03 (0.98, 1.06)
Natural indirect association	**1.02 (1.02, 1.03)**	**1.04 (1.03, 1.04)**	**1.03 (1.02, 1.04)**	**1.01 (1.00, 1.01)**
Proportion mediated,%	29.8	23.9	23.3	19.0
**Alcohol consumption**^d^				
Natural direct association	**1.07 (1.01, 1.12)**	**1.15 (1.11, 1.23)**	**1.15 (1.09, 1.24)**	1.03 (0.98, 1.06)
Natural indirect association	**1.01 (1.01, 1.02)**	**1.01 (1.01, 1.01)**	**1.00 (1.00, 1.01)**	**1.00 (1.00, 1.01)**
Proportion mediated,%	17.5	8.2	2.4	15.4
**Physical activity**^e^				
Natural direct association	**1.08 (1.06, 1.11)**	**1.15 (1.10, 1.21)**	**1.13 (1.03, 1.21)**	1.03 (0.98, 1.08)
Natural indirect association	**1.01 (1.01, 1.01)**	**1.01 (1.01, 1.02)**	**1.01 (1.00, 1.02)**	**1.00 (1.00, 1.00)**
Proportion mediated,%	9.3	9.1	8.0	9.8
**Lifestyle behaviours (smoking status, alcohol consumption, and physical activity)**				
Natural direct association	**1.05 (1.02, 1.08)**	**1.11 (1.05, 1.17)**	**1.10 (1.01, 1.19)**	1.02 (0.97, 1.05)
Natural indirect association	**1.03 (1.03, 1.04)**	**1.06 (1.05, 1.07)**	**1.04 (1.03, 1.05)**	**1.01 (1.01, 1.02)**
Proportion mediated,%	41.0	38.0	30.5	41.8
**All four**				
Natural direct association	0.99 (0.97, 1.04)	1.04 (0.99, 1.08)	1.05 (0.97, 1.11)	0.97 (0.92, 1.02)
Natural indirect association	**1.07 (1.06, 1.08)**	**1.12 (1.11, 1.15)**	**1.09 (1.07, 1.12)**	**1.04 (1.03, 1.05)**
Proportion mediated,%	86.8	74.1	65.1	122.6
	**Number of pain sites**			
	**One**	**Two**	**Three**	**Four**
Total association	**1.09 (1.06, 1.12)**	**1.25 (1.21, 1.30)**	**1.43 (1.36, 1.51)**	**1.46 (1.35, 1.57)**
**Opioid use**				
Natural direct association	**1.03 (1.01, 1.06)**	**1.17 (1.14, 1.21)**	**1.30 (1.22, 1.36)**	**1.11 (1.03, 1.20)**
Natural indirect association	**1.04 (1.03, 1.05)**	**1.08 (1.07, 1.10)**	**1.11 (1.08, 1.14)**	**1.32 (1.24, 1.41)**
Proportion mediated,%	47.0	34.9	32.5	45.2
**Smoking status**				
Natural direct association	**1.07 (1.04, 1.11)**	**1.22 (1.18, 1.27)**	**1.38 (1.31, 1.46)**	**1.38 (1.29, 1.48)**
Natural indirect association	**1.02 (1.02, 1.03)**	**1.04 (1.03, 1.04)**	**1.05 (1.04, 1.06)**	**1.08 (1.06, 1.11)**
Proportion mediated,%	24.7	17.1	14.6	22.2
**Alcohol consumption**				
Natural direct association	**1.08 (1.06, 1.11)**	**1.23 (1.19, 1.26)**	**1.39 (1.35, 1.47)**	**1.40 (1.29, 1.48)**
Natural indirect association	**1.01 (1.01, 1.01)**	**1.02 (1.01, 1.02)**	**1.04 (1.03, 1.06)**	**1.04 (1.01, 1.08)**
Proportion mediated,%	10.5	8.7	13.0	12.7
**Physical activity**				
Natural direct association	**1.08 (1.06, 1.11)**	**1.23 (1.18, 1.27)**	**1.39 (1.32, 1.46)**	**1.40 (1.30, 1.52)**
Natural indirect association	**1.01 (1.01, 1.01)**	**1.01 (1.01, 1.02)**	**1.04 (1.03, 1.04)**	**1.05 (1.03, 1.08)**
Proportion mediated,%	9.1	9.8	11.8	15.7
**Lifestyle behaviours (smoking status, alcohol consumption, and physical activity)**				
Natural direct association	**1.06 (1.03, 1.09)**	**1.18 (1.14, 1.22)**	**1.31 (1.27, 1.36)**	**1.28 (1.20, 1.38)**
Natural indirect association	**1.04 (1.03, 1.04)**	**1.07 (1.06, 1.08)**	**1.12 (1.10, 1.13)**	**1.17 (1.13, 1.20)**
Proportion mediated,%	39.7	31.1	34.2	42.8
**All four**				
Natural direct association	1.01 (0.97, 1.04)	**1.07 (1.01, 1.11)**	**1.10 (1.03, 1.17)**	1.00 (0.92, 1.10)
Natural indirect association	**1.07 (1.06, 1.08)**	**1.13 (1.11, 1.14)**	**1.20 (1.16, 1.23)**	**1.30 (1.23, 1.39)**
Proportion mediated,%	73.7	54.1	53.4	69.6

Data are presented as hazard ratio (95% confidence interval) unless otherwise indicated. Effect estimates with statistical significance are labelled in bold.

a The effect estimate with its 95% confidence interval was slightly different for each mediation analysis. To reduce overlap, we listed the value from Tables 2 and 3.

b The mediator of opioid use was modelled as yes vs no.

c The mediator of smoking status was modelled as current smoking vs never or previous smoking.

d The mediator of alcohol consumption was modelled as regular vs special occasions or never.

e The mediator of physical activity was modelled as low vs moderate or high.

## 5 Discussion

In a large population of middle-aged UK participants, neck or shoulder pain only, back pain only and knee pain only were associated with increased risk of all-cause mortality. Participants with higher number of pain sites had increased risk of all-cause mortality. However, these associations were mediated by physical activity, smoking status, alcohol consumption, and opioid use. At least half of the association of chronic musculoskeletal pain with increased all-cause mortality may be accounted for by four mediators.

The results of our all-cause mortality analyses are consistent with those of previous studies [3,6]. In contrast to previous studies, we clearly defined chronic musculoskeletal pain using two specific questions in UK Biobank. In addition, we were able to choose an appropriate comparison group by excluding participants with other types of pain and non-chronic musculoskeletal pain and assess the influence from the type of pain by focusing on those with one pain site. Considering number of pain sites, our results were similar to those of a recent Danish study [2], however the definition of musculoskeletal pain differs (ours: chronic vs the Danish study: pain in the last 14 days). Thus, the similar results might indicate a higher mortality risk for those with pain at multiple sites, compared to people with pain in one site, irrespective of the duration of pain. For participants experiencing pain in two or more sites, the results from our exploratory analysis which indicated that younger participants had higher risk of all-cause mortality, might reflect immortal time bias, as older participants might have been healthier enough to live longer at study entry compared to younger participants [29]. Considering cause-specific mortality analyses, our results are in line with previous research [2,3]. However, previous studies had smaller samples, yielding less precise estimates; they also did not consider the competing risk in analysing cause-specific mortality, which might have biased results [2,3,30]. We have a large sample size and used a multistate survival model, which allowed us to confirm that the association between respiratory system disease mortality or digestive system disease mortality was stronger than that of all-cause mortality among participants with two or more pain sites [25]. This might indicate that better management is needed for respiratory system disease or digestive system disease among participants with chronic musculoskeletal pain in two or more sites.

For the mediator - physical activity and alcohol consumption, our results are consistent with a previous study [6]. For the mediator - smoking status, our results showed that the relationship between chronic musculoskeletal pain and all-cause mortality was mediated by smoking status, which is in contrast to previous findings [6]. This difference might be due to the small sample size in the previous study (6324 vs ours: 384,367), which makes it difficult to detect moderate associations [6]. The results from our multiple mediator analyses indicated that most of the association between chronic musculoskeletal pain and all-cause mortality were mediated by all four factors, which means that poor lifestyle factors and opioid use may be the main drivers that increased the risk of all-cause mortality, rather than chronic musculoskeletal pain. Current guidelines indicate that: 1. people with chronic pain should remain physically active; 2. opioids should be avoided in general and only be used when the benefits outweigh the potential risks if other options fail [31], [32], [33]. Our study contributed to the field by providing more comprehensive and accurate results. Additionally, the results indicated that smoking cessation and alcohol consumption control should be added in future guidelines. Although educating patients to use less opioids is important, it can be challenging. Meanwhile, some people might need to use opioid if other therapies fail. One guideline mention that opioids should always be combined with nonpharmacologic and often nonopioid pharmacologic therapy [33], however, no specific guidance is provided. Our results showed that keeping adequate levels of physical activity, smoking cessation and alcohol consumption controlled should be emphasised to people who take opioids, as these healthy lifestyle behaviours could substantially decrease mortality risk.

To our knowledge, this is the first large population-based study to comprehensively assess the association between chronic musculoskeletal pain (type of pain and number of pain sites) and mortality (all-cause and cause-specific mortality). Further, it is also the first study to document that the association is mediated by lifestyle factors and opioid use, individually and simultaneously. Several additional analyses were performed to confirm the robustness of the results.

Some limitations need consideration. First, data on pain intensity and pain-related symptoms (e.g., numbness and itching) were not included in this study. Likewise, we did not have any data on the actual duration of symptoms and, therefore, could not ascertain the role of pain duration on the association between chronic musculoskeletal pain and mortality. Although there is no evidence that these factors could bias our results, future studies should untangle their roles. Second, the UK Biobank collected data from UK participants with specific ages (40–69), and we must exercise caution when generalising these findings to other age groups or other countries. Third, number of events in some categories for cause-specific mortality (e.g. falls and suicide) may be too small, and we caution the reader in making inferences based on these imprecise results. Fourth, the dose and duration of opioid use was not included so that overdose death could not be assessed. We defined opioid use through regular treatments taken weekly, monthly, etc. (short-term use was not included). Future studies should include data with doses, formulations and prescription dates (e.g. primary care data) to provide more accurate results. Fifth, due to the study scope, other types of chronic pain (e.g. stomach or abdominal pain) were not included. Assuming the definition of pain in the UK Biobank included eight different presentations (i.e. headache, facial pain, neck or shoulder pain, back pain, stomach or abdominal pain, hip pain, knee pain and pain all over the body), participants would have a total of 255 ($C_{8}^{1}+C_{8}^{2}+C_{8}^{3}+C_{8}^{4}+C_{8}^{5}+C_{8}^{6}+C_{8}^{7}+C_{8}^{8}$) possible combinations considering pain status. Our analyses would be arguably underpowered if we were to include all the possible combinations. Sixth, insufficient primary care data in the current study makes it difficult to identify accurate cases of specific musculoskeletal pain diagnoses (e.g. autoimmune/rheumatic diseases) which could provide new insights in understanding the association between chronic musculoskeletal pain and mortality. The UK Biobank plans to release primary care data for all participants in future, providing the opportunity to further explore this issue. Seventh, we acknowledge certain limitations in the measurement of alcohol consumption and physical activity (ie. subjective measurement of alcohol consumption and physical activity; and qualitative measurement of alcohol consumption) could have biased the results. Objective and more comprehensive measurement should be explored in future studies. Finally, missing data in the mediators might have affected the results despite our approach of multiple imputation to handle this issue.

Higher number of pain sites was associated with increased risk of all-cause mortality compared to having no pain, and at least half of the association of chronic musculoskeletal pain with increased all-cause mortality may be accounted for by four mediators. Supporting healthy lifestyle behaviour (keeping adequate levels of physical activity, smoking cessation and alcohol consumption controlled) as well as opioids deprescription is an important strategy to decrease the mortality risk associated with chronic musculoskeletal pain.

## Funding

Twins Research Australia.

## Authors’ contributions

All authors designed the study. LC conducted the data analysis and drafted the manuscript. All authors critically revised the manuscript for important intellectual content. All authors approved the final version of the manuscript. LC and PHF verified the underlying data reported in the manuscript. The corresponding author attests that all the listed authors meet authorship criteria and that no others meeting the criteria have been omitted.

## Data sharing statement

Data from UK Biobank are available on application at www.ukbiobank.ac.uk/register-apply.

## Declaration of Competing Interest

Dr Arden reported receiving personal fees from Pfizer/Lilly and Bristows LLP and grants from Merck outside the submitted work. Dr Nassar is supported by Financial Markets for Children and NHMRC. No other disclosures were reported.

## Appendix Supplementary materials

Image, application 1
