# Supplementary material for: Association of chronic musculoskeletal pain with mortality among UK adults: A population-based cohort study with mediation analysis
Source: eClinicalMedicine. 2021 Nov 14;42:101202. doi: 10.1016/j.eclinm.2021.101202 (PMC8605211; doi:10.1016/j.eclinm.2021.101202)
Supplement: Supplementary file 1 [file mmc1.docx]

**Appendix**

|  | Page |
| --- | --- |
| Appendix S1. Details for opioids. | 2 |
| Appendix S2. Methods for exploratory and sensitivity analyses. | 3 |
| Appendix S3. Results for exploratory analyses. | 4-6 |
| Appendix S4. Results for sensitivity analyses: induction period. | 7-9 |
| Appendix S5. Results for sensitivity analyses: other covariates. | 10 |
| Appendix S6. Results for sensitivity analyses: exclude cancer patients. | 11 |
| Appendix S7. Results for sensitivity analyses: E-value. | 12 |
| Appendix S8. Transition probabilities. | 13 |

**Appendix S1. Details for opioids.**

| Name | Code |
| --- | --- |
| Tramadol | 1140864992 |
| Paracetamol + Tramadol | 1141190956 |
| Codeine | 1140884444 |
| Dihydrocodeine | 1140884464 |
| Medocodeine Tablet | 1140856406 |
| Ibuprofen + Codeine Phosphate | 1140878030 |
| Aspirin + Codeine 300mg/8mg tablet | 1140882268 |
| Aspirin + Codeine | 1140882392 |
| Pracetamol + Codeine | 1140882394 |
| Paracetamol + Dihydrocodeine tartrate | 1140882396 |
| Codeine phosphate + Kaolin 10mg/3g/10ml mixture | 1140865654 |
| Dihydrocodeine | 1140884464 |
| Morphine | 1140871692 |
| kaolin+morphine | 1140882114 |
| morphine sulphate+atropine sulphate | 1140882116 |
| morphine tartrate+cyclizine | 1140882406 |
| diamorphine | 1140884460 |
| methylmorphine | 1140910376 |
| diacetylmorphine | 1140910402 |
| oxycodone hydrochloride | 1141171038 |
| fentanyl+droperidol | 1140879212 |
| fentanyl | 1140880956 |
| fentanyl product | 1141157470 |
| pethidine | 1140884388 |
| methadone | 1140884482 |
| martindale methadone dtf 1mg/ml mixture | 1140922628 |
| heroin | 1140888836 |
| buprenorphine | 1140871732 |
| co-codamol | 1140923346 |
| co-proxamol | 1140923348 |
| co-dydramol | 1140923350 |
| co-codaprin | 1140923344 |

**Appendix S2.** **Methodology for** **exploratory and sensitivity analyses.**

Exploratory analyses

Based on previous literature and clinical knowledge, we examined whether the association between chronic musculoskeletal pain and all-cause mortality differed by sex, age, BMI, ethnicity, or smoking status through testing of multiplicative interactions using WALD statistics (Harrell Jr, Frank E. *Regression modeling strategies: with applications to linear models, logistic and ordinal regression, and survival analysis*. Springer, 2015.). To avoid potential multiple testing issue, we chose the number of pain sites as the exposure and treated it as an unordered categorical variable.

Sensitivity analyses

1. To assess the influence of potential induction period, we used different lag time periods (1, 3, 5, and 7-year lag). Exposure status at a given time will correlate with a possible increase or decrease in disease only at some later time, which might introduce bias if we modelled the exposure-outcome association without considering this issue; lag period analysis could assess the potential influence by the induction period (Lash TL, VanderWeele TJ, Haneause S, Rothman K. *Modern epidemiology.* Lippincott Williams & Wilkins; 2020).

2. Except the covariates adjusted, there are other covariates which might be considered as confounders. However, the relationship between these covariates and the exposure might be bi-directional. Thus, we performed a sensitivity analysis including these covariates: body mass index (continuous), diabetes (yes or no), cancer (yes or no), depression (yes or no), anxiety (yes or no), cardiovascular disease (included heart attack, angina, stroke and high blood pressure; codes as the number of cardiovascular disease; the value ranged from 0-4). Two models were used: outcome regression and inverse probability treatment weighting through twang package. With twang package, gradient boosted models (number of tress was 5000 and 2000 for the analysis of pain type and number of pain sites, respectively) were used to calculate propensity score.

3. Severe cancer patients could have severe pain, which might bias the results. We could not identify severe cancer patients. Thus, we excluded participants with cancer.

4. To explore the potential influence from unmeasured confounding, E-value was calculated (VanderWeele TJ, Ding P. Sensitivity Analysis in Observational Research: Introducing the E-Value. *Annals of internal medicine.* 2017;167(4):268-274.).

In the main analyses, results from model 2 (analyses adjusted for age, sex, ethnicity, and the Townsend deprivation index) are reported in the results section. To increase readability for the above exploratory and sensitivity analyses, results from model 2 are presented in Appendix S4-S8.

**Appendix S3. Results for exploratory analyses.**

We found the association between chronic musculoskeletal pain and all-cause mortality differed by age. Considering the data distribution and clinical meaning, we chose 60 as the cut-off point. We also set 55 as another cut-off point to verify the results.

|  | **Pain type** | | | | |
| --- | --- | --- | --- | --- | --- |
|  | **No pain** | **Neck or shoulder pain** | **Back pain** | **Hip Pain** | **Knee Pain** |
| >= 60 |  |  |  |  |  |
| No of deaths (n=14159) | 8670 | 1399 | 1625 | 593 | 1872 |
| Multivariable adjusted^a^ | 1 (reference) | 1.07 (1.01, 1.14) | 1.16 (1.10, 1.23) | 1.14 (1.05, 1.24) | 1.01 (0.96, 1.06) |
| < 60 |  |  |  |  |  |
| No of deaths (n=5273) | 3207 | 558 | 680 | 167 | 661 |
| Multivariable adjusted^a^ | 1 (reference) | 1.07 (0.98, 1.17) | 1.16 (1.07, 1.26) | 1.17 (1.00, 1.36) | 1.10 (1.01, 1.19) |
| >= 55 |  |  |  |  |  |
| No of deaths (n=16801) | 10281 | 1687 | 1941 | 687 | 2205 |
| Multivariable adjusted^a^ | 1 (reference) | 1.09 (1.03, 1.14) | 1.17 (1.11, 1.22) | 1.16 (1.07, 1.25) | 1.02 (0.97, 1.07) |
| < 55 |  |  |  |  |  |
| No of deaths (n=2631) | 1596 | 270 | 364 | 73 | 328 |
| Multivariable adjusted^a^ | 1 (reference) | 0.99 (0.87, 1.13) | 1.15 (1.03, 1.29) | 1.07 (0.85, 1.36) | 1.14 (1.01, 1.28) |
|  | **Number of pain sites** | | | | |
|  | **No Pain** | **One** | **Two** | **Three** | **Four** |
| >= 60 years |  |  |  |  |  |
| No of deaths (n=18832) | 8670 | 5489 | 2890 | 1310 | 473 |
| Multivariable adjusted^a^ | 1 (reference) | 1.08 (1.04, 1.12) | 1.22 (1.17, 1.28) | 1.36 (1.28, 1.44) | 1.34 (1.22, 1.47) |
| < 60 years |  |  |  |  |  |
| No of deaths (n=7085) | 3207 | 2066 | 1059 | 537 | 216 |
| Multivariable adjusted^a^ | 1 (reference) | 1.11 (1.05, 1.18) | 1.32 (1.23, 1.42) | 1.65 (1.51, 1.81) | 1.82 (1.59, 2.09) |
| >= 55 years |  |  |  |  |  |
| No of deaths (n=22436) | 10281 | 6520 | 3429 | 1599 | 607 |
| Multivariable adjusted^a^ | 1 (reference) | 1.09 (1.06, 1.12) | 1.24 (1.19, 1.29) | 1.40 (1.33, 1.48) | 1.44 (1.33, 1.57) |
| < 55 years |  |  |  |  |  |
| No of deaths (n=3481) | 1596 | 1035 | 520 | 248 | 82 |
| Multivariable adjusted^a^ | 1 (reference) | 1.09 (1.01, 1.18) | 1.33 (1.20, 1.47) | 1.71 (1.49, 1.95) | 1.60 (1.28, 2.00) |

Data are presented as hazard ratio (95% confidence interval) unless otherwise indicated.

^a^ Adjusted for age, sex, townsend deprivation index and ethnicity.

**Appendix S4. Results for sensitivity analyses: induction period.**

|  | **Pain type** | | | | |
| --- | --- | --- | --- | --- | --- |
|  | **No pain** | **Neck or shoulder pain** | **Back pain** | **Hip Pain** | **Knee Pain** |
| 3 months |  |  |  |  |  |
| No of deaths (n=19359) | 11834 | 1949 | 2293 | 757 | 2526 |
| Multivariable adjusted^a^ | 1 (reference) | 1.07 (1.02, 1.13) | 1.16 (1.11, 1.22) | 1.15 (1.07, 1.24) | 1.03 (0.99, 1.08) |
| 6 months |  |  |  |  |  |
| No of deaths (n=19221) | 11746 | 1935 | 2275 | 752 | 2513 |
| Multivariable adjusted^a^ | 1 (reference) | 1.07 (1.02, 1.13) | 1.16 (1.11, 1.22) | 1.15 (1.07, 1.24) | 1.04 (0.99, 1.08) |
| 1 year |  |  |  |  |  |
| No of deaths (n=18909) | 11557 | 1894 | 2234 | 744 | 2480 |
| Multivariable adjusted^a^ | 1 (reference) | 1.07 (1.02, 1.12) | 1.16 (1.11, 1.22) | 1.16 (1.07, 1.25) | 1.04 (0.99, 1.08) |
| 3 years |  |  |  |  |  |
| No of deaths (n=16966) | 10395 | 1685 | 1989 | 665 | 2232 |
| Multivariable adjusted^a^ | 1 (reference) | 1.06 (1.00, 1.11) | 1.15 (1.10, 1.21) | 1.15 (1.06, 1.24) | 1.04 (0.99, 1.09) |
| 5 years |  |  |  |  |  |
| No of deaths (n=14444) | 8810 | 1447 | 1677 | 568 | 1942 |
| Multivariable adjusted^a^ | 1 (reference) | 1.07 (1.01, 1.13) | 1.15 (1.09, 1.21) | 1.16 (1.06, 1.26) | 1.07 (1.02, 1.12) |
| 7 years |  |  |  |  |  |
| No of deaths (n=11204) | 6805 | 1139 | 1299 | 447 | 1514 |
| Multivariable adjusted^a^ | 1 (reference) | 1.08 (1.02, 1.15) | 1.15 (1.08, 1.22) | 1.18 (1.07, 1.30) | 1.08 (1.02, 1.14) |
|  | **Number of pain sites** | | | | |
|  | **No Pain** | **One** | **Two** | **Three** | **Four** |
| 3 months |  |  |  |  |  |
| No of deaths (n=25817) | 11834 | 7525 | 3929 | 1842 | 687 |
| Multivariable adjusted^a^ | 1 (reference) | 1.09 (1.06, 1.12) | 1.25 (1.20, 1.30) | 1.43 (1.37, 1.51) | 1.46 (1.35, 1.58) |
| 6 months |  |  |  |  |  |
| No of deaths (n=25632) | 11746 | 7475 | 3895 | 1832 | 684 |
| Multivariable adjusted^a^ | 1 (reference) | 1.09 (1.06, 1.12) | 1.25 (1.20, 1.29) | 1.44 (1.37, 1.51) | 1.46 (1.35, 1.58) |
| 1 year |  |  |  |  |  |
| No of deaths (n=25216) | 11557 | 7352 | 3825 | 1805 | 677 |
| Multivariable adjusted^a^ | 1 (reference) | 1.09 (1.06, 1.12) | 1.24 (1.20, 1.29) | 1.44 (1.37, 1.51) | 1.47 (1.36, 1.59) |
| 3 years |  |  |  |  |  |
| No of deaths (n=22619) | 10395 | 6571 | 3420 | 1619 | 614 |
| Multivariable adjusted^a^ | 1 (reference) | 1.08 (1.05, 1.12) | 1.24 (1.19, 1.29) | 1.43 (1.36, 1.51) | 1.48 (1.37, 1.61) |
| 5 years |  |  |  |  |  |
| No of deaths (n=19280) | 8810 | 5634 | 2900 | 1404 | 532 |
| Multivariable adjusted^a^ | 1 (reference) | 1.09 (1.06, 1.13) | 1.24 (1.18, 1.29) | 1.47 (1.39, 1.55) | 1.52 (1.39, 1.66) |
| 7 years |  |  |  |  |  |
| No of deaths (n=14996) | 6805 | 4399 | 2258 | 1119 | 415 |
| Multivariable adjusted^a^ | 1 (reference) | 1.10 (1.06, 1.15) | 1.24 (1.18, 1.30) | 1.51 (1.42, 1.61) | 1.52 (1.38, 1.68) |

Data are presented as hazard ratio (95% confidence interval) unless otherwise indicated.

^a^ Adjusted for age, sex, townsend deprivation index and ethnicity.

Appendix S5. Results for sensitivity analyses: other covariates.

|  | **Pain type** | | | | |
| --- | --- | --- | --- | --- | --- |
|  | **No pain** | **Neck or shoulder pain** | **Back pain** | **Hip Pain** | **Knee Pain** |
| No of deaths (n=19441) | 11877 | 1957 | 2305 | 769 | 2533 |
|  |  |  |  |  |  |
| Outcome regression | 1 (reference) | 1.05 (1.00, 1.10) | 1.12 (1.07, 1.17) | 1.09 (1.01, 1.18) | 0.97 (0.93, 1.02) |
|  |  |  |  |  |  |
| IPTW | 1 (reference) | 1.06 (1.01, 1.11) | 1.10 (1.05, 1.15) | 1.09 (1.00, 1.18) | 0.99 (0.94, 1.04) |
|  | **Number of pain sites** | | | | |
|  | **No Pain** | **One** | **Two** | **Three** | **Four** |
| No of deaths (n=25917) | 11877 | 7555 | 3949 | 1847 | 689 |
|  |  |  |  |  |  |
| Outcome regression | 1 (reference) | 1.05 (1.02, 1.08) | 1.15 (1.11, 1.19) | 1.25 (1.19, 1.32) | 1.21 (1.11, 1.30) |
|  |  |  |  |  |  |
| IPTW | 1 (reference) | 1.05 (1.02, 1.08) | 1.15 (1.11, 1.20) | 1.29 (1.22, 1.36) | 1.28 (1.16, 1.41) |

IPTW: inverse probability treatment weighting. Data are presented as hazard ratio (95% confidence interval) unless otherwise indicated.

Included covariates in both models: age, sex, townsend deprivation index, ethnicity, body mass index, diabetes, cancer, depression, anxiety, and cardiovascular disease.

Appendix S6. Results for sensitivity analyses: exclude cancer patients.

|  | **Pain type** | | | | |
| --- | --- | --- | --- | --- | --- |
|  | **No pain** | **Neck or shoulder pain** | **Back pain** | **Hip Pain** | **Knee Pain** |
| No of deaths (n=16084) | 9865 | 1598 | 1875 | 608 | 2138 |
| Multivariable adjusted^a^ | 1 (reference) | 1.06 (1.00, 1.11) | 1.14 (1.09, 1.20) | 1.13 (1.04, 1.23) | 1.05 (1.00, 1.10) |
|  | **Number of pain sites** | | | | |
|  | **No Pain** | **One** | **Two** | **Three** | **Four** |
| No of deaths (n=21395) | 9865 | 6219 | 3221 | 1521 | 569 |
| Multivariable adjusted^a^ | 1 (reference) | 1.08 (1.05, 1.12) | 1.24 (1.20, 1.30) | 1.44 (1.37, 1.52) | 1.49 (1.37, 1.63) |

Data are presented as hazard ratio (95% confidence interval) unless otherwise indicated.

^a^ Adjusted for age, sex, townsend deprivation index and ethnicity.

Appendix S7. Results for sensitivity analyses: E-value.

| **Pain type** | | | | |
| --- | --- | --- | --- | --- |
| **No pain** | **Neck or shoulder pain** | **Back pain** | **Hip Pain** | **Knee Pain** |
| 1 (reference) | 1.34 (1.16) | 1.62 (1.46) | 1.57 (1.34) | 1.21 (1) |
| **Number of pain sites** | | | | |
| **No Pain** | **One** | **Two** | **Three** | **Four** |
| 1 (reference) | 1.4 (1.31) | 1.81 (1.71) | 2.21 (2.06) | 2.28 (2.04) |

Data are presented as hazard ratio (95% confidence interval) unless otherwise indicated.

Appendix S8. Transition probabilities.

|  | Neck or shoulder pain only | Back pain only | Hip pain only | Knee pain only | Number of pain sites (whole cohort) |
| --- | --- | --- | --- | --- | --- |
| Survival | 0.9394385 | 0.9385606 | 0.9390286 | 0.9384451 | 0.9325723 |
| Death due to cancer | 0.03284171 | 0.03324539 | 0.03324311 | 0.03333191 | 0.03509146 |
| Death due to cardiovascular disease | 0.0114784 | 0.01161466 | 0.0116182 | 0.01175566 | 0.01306304 |
| Death due to mental and behavioural disorder | 0.001221386 | 0.001303996 | 0.001215858 | 0.001260145 | 0.001412712 |
| Death due to respiratory system disease | 0.003813001 | 0.003911987 | 0.003797145 | 0.003797522 | 0.004826117 |
| Suicide | 0.0005121942 | 0.0005285298 | 0.0005162573 | 0.0005040581 | 0.0005099293 |
| Death due to nervous system disease | 0.003322695 | 0.00333147 | 0.003333961 | 0.003314823 | 0.003611132 |
| Death due to endocrine, nutritional and metabolic disease | 0.0006260151 | 0.0005848485 | 0.0005645056 | 0.0005467749 | 0.0007674956 |
| Death due to digestive system disease | 0.001996244 | 0.00201881 | 0.001944408 | 0.002114481 | 0.002515825 |
| Death due to musculoskeletal system and connective tissue disease | 0.0001794868 | 0.0001949495 | 0.0001688692 | 0.0001922255 | 0.0002445579 |
| Death due to genitourinary system disease | 0.0003327073 | 0.0003292481 | 0.0003184391 | 0.0003289193 | 0.0004136671 |
| Death due to fallls | 0.0004290173 | 0.000472211 | 0.0004390599 | 0.0004442546 | 0.0004630991 |
| Death due to other causes | 0.003808623 | 0.003903322 | 0.003811619 | 0.003964118 | 0.004508712 |
